# Autistic traits in unaffected first-degree relatives of individuals with schizophrenia and bipolar disorder: implications for shared familial neurodevelopmental vulnerability

**DOI:** 10.3389/fpsyt.2026.1933974

**Published:** 2026-09-09

**Authors:** Fatma Büşra Parlakkaya Yıldız, Ayşe Kurtulmuş, Zülal Çelik, Beyzanur Ereğli, Aynur Görmez

**Affiliations:** Department of Psychiatry, Faculty of Medicine, Istanbul Medeniyet University, Istanbul, Türkiye

**Keywords:** autistic traits, bipolar disorder, broader autism phenotype, first-degree relatives, neurodevelopmental vulnerability, schizophrenia

## Abstract

**Background:**

Autistic traits have increasingly been linked to schizophrenia (SCH) and bipolar disorder (BD) within a shared neurodevelopmental framework. However, whether these traits are similarly expressed in unaffected first-degree relatives of individuals with SCH and BD remains poorly understood.

**Methods:**

This cross-sectional study included 135 participants: first-degree relatives of individuals with schizophrenia (SCH-FDR, n=45), first-degree relatives of individuals with bipolar disorder (BD-FDR, n=45), and healthy controls (HCs, n=45). Autistic traits were assessed using the Autism Spectrum Quotient (AQ). Participants also underwent clinical psychiatric evaluation, including structured diagnostic assessment, to exclude psychiatric disorders and autism spectrum disorder.

**Results:**

Significant group differences were observed for AQ total score (p=0.002), Communication (p=0.004), and Imagination (p<0.001) subscales. Communication scores were significantly higher in SCH-FDR than in HCs (p=0.003). Imagination scores were higher in both BD-FDR (p=0.016) and SCH-FDR (p<0.001) groups compared with HCs. Total AQ scores were also higher in both relative groups than in HCs (BD-FDR: p=0.015; SCH-FDR: p=0.003). The proportion of participants with AQ scores ≥26 was higher in SCH-FDR (17.8%) and BD-FDR (13.3%) than in HCs (2.2%; p=0.045).

**Conclusions:**

Autistic traits were more pronounced in SCH-FDR and BD-FDR than in healthy controls. Differences were most evident in total AQ scores and the Imagination subscale across both relative groups, whereas communication difficulties were specifically elevated in schizophrenia relatives. These findings are consistent with the concept of shared familial neurodevelopmental vulnerability across schizophrenia and bipolar disorder.

## 1 Introduction

Schizophrenia (SCH) and bipolar disorder (BD) are severe psychiatric conditions with markedly different clinical presentations, yet accumulating genomic and epidemiological findings suggest they share a common neurodevelopmental background. The neurodevelopmental hypothesis originally formulated for schizophrenia has been considerably broadened over the past two decades to encompass a gradient of conditions with overlapping biological and clinical features, within which autism spectrum disorder (ASD) occupies a central position (1–4).

ASD is defined by persistent impairments in social communication and interaction alongside restricted, repetitive patterns of behavior, with symptoms present from early development (5). Beyond its core diagnostic features, ASD has also been linked to schizophrenia spectrum disorders and BD across several domains, including clinical features, cognition, familial aggregation, and underlying biology (6–8).

The neurodevelopmental continuum model proposes that SCH and BD, rather than being etiologically discrete entities, lie along a gradient of shared neurodevelopmental liability within which ASD also participates. This framework is supported by convergent evidence from family-based, epidemiological, and genomic studies demonstrating overlap among ASD, SCH, and BD across diagnostic boundaries (1–4, 7, 8).

Within this neurodevelopmental framework, autistic traits have increasingly been conceptualized as continuous, dimensional characteristics distributed across the general population, rather than features confined to categorical ASD diagnoses (9). Elevated levels of autistic traits have been consistently reported in individuals with SCH compared with HCs, and meta-analytic evidence supports their presence across schizophrenia spectrum disorders (10–12). Moreover, autistic traits in SCH have been associated with poorer social cognition, non-social cognition, and everyday functioning, even after accounting for other clinical variables (13, 14). Similar observations have been reported in BD, where subclinical autistic traits have been linked to impairments in social cognition, emotion regulation, and broader psychosocial functioning (15–17).

Increasing attention has also been directed toward first-degree relatives of individuals with these conditions, in whom autistic traits may be present in the absence of a diagnosable disorder. A multicenter Italian study (NIRP) of 342 unaffected first-degree relatives of individuals with schizophrenia spectrum disorders found that 44.9% presented with autistic symptoms, and higher symptom levels were associated with poorer working memory, social cognition, and functional outcomes (18). Evidence regarding autistic traits in first-degree relatives of individuals with BD remains relatively limited. However, social-cognitive alterations have been reported in unaffected relatives of individuals with bipolar disorder, and meta-analytic findings indicate impaired Theory of Mind performance compared with healthy controls (19).

Whether autistic traits represent a shared characteristic of familial liability across SCH and BD, and whether any such overlap involves autistic traits broadly or particular trait dimensions, remains unclear. Despite this growing evidence, studies directly comparing autistic trait profiles across familial risk groups for both SCH and BD within the same study design remain scarce. The present study aimed to assess and compare autistic traits, including total and subdomain-level AQ scores, in first-degree relatives of individuals with schizophrenia and bipolar disorder and in healthy controls. By directly comparing unaffected first-degree relatives of individuals with schizophrenia and bipolar disorder within the same study, it sought to contribute to a better understanding of whether autistic traits show similar or distinct patterns across familial risk groups within the neurodevelopmental continuum framework.

## 2 Materials and methods

### 2.1 Study design and participants

This cross-sectional study was conducted between June 2024 and June 2025 in the outpatient psychiatry clinics of Istanbul Medeniyet University Göztepe Prof. Dr. Süleyman Yalçın City Hospital. The study sample consisted of first-degree relatives (FDR) of patients with SCH and BD who were under regular follow-up in these clinics.

Participants aged 18–65 years were included if they were first-degree relatives of patients with SCH or BD and provided written informed consent. Individuals with any history of psychiatric consultation, psychiatric diagnosis, or psychotropic medication use were excluded.

All participants underwent a comprehensive psychiatric evaluation. Lifetime psychiatric disorders were assessed using the Structured Clinical Interview for DSM-5 (SCID-5), and individuals meeting DSM-5 criteria for any psychiatric disorder were excluded. In addition, participants were clinically evaluated for autism spectrum disorder according to DSM-5 criteria, as there is no validated clinician-rated autism instrument for adults in Turkiye. Developmental history relevant to autism spectrum disorder, including childhood social communication, language development, and restricted or repetitive behaviors, was obtained from both the participant and an accompanying informant during the clinical assessment. None of the included participants met DSM-5 diagnostic criteria for autism spectrum disorder.

A healthy control (HC) group was recruited from volunteers with no personal or first-degree family history of psychiatric illness. During recruitment, we aimed to achieve comparable age and sex distributions across the three groups; however, individual matching was not performed. Exclusion criteria for all participants included neurological disease, substance use disorder, major medical illness, or inability to complete the assessment procedures.

### 2.2 Ethics statement

The study protocol was approved by the Ethics Committee of Istanbul Medeniyet University Göztepe Prof. Dr. Süleyman Yalçın City Hospital (Decision No: 2023/0460) and was conducted in accordance with the principles of the Declaration of Helsinki. Written informed consent was obtained from all participants prior to participation.

### 2.3 Measures

#### 2.3.1 Autism Spectrum Quotient

Autistic traits were assessed using the Autism Spectrum Quotient (AQ), a 50-item self-report questionnaire developed by Baron-Cohen and colleagues to measure autistic-like characteristics in adults of normal intelligence (20). The AQ is one of the most widely used and cross-culturally validated self-report measures of autistic traits, with demonstrated ability to differentiate between autistic and non-autistic individuals across diverse clinical and nonclinical populations. Items are scored in a binary format (agree/disagree), and higher scores reflect a higher degree of autistic-like characteristics (21–23). Both total AQ scores and subscale scores were included in the analyses. An AQ score of ≥26 was used to indicate clinically elevated autistic traits (22, 24).

The scale comprises five theoretically derived subdomains of 10 items each:

Social Skills measures difficulties in social interaction, social reciprocity, and the ability to engage spontaneously with others. Higher scores reflect reduced capacity to initiate and maintain social relationships.

Attention Switching captures difficulties in flexibly shifting attention between tasks or perspectives.

Attention to Detail assesses a tendency to focus on specific details rather than the gestalt.

Communication evaluates difficulties in pragmatic and non-verbal communication, including challenges with conversational reciprocity, understanding implied meaning, and non-literal language.

Imagination assesses the capacity for flexible, generative imagining, including the ability to invent stories, imagine alternative scenarios, and engage spontaneously with fictional or creative content (21).

The Turkish version of the AQ has previously demonstrated acceptable validity and reliability (24, 25). The AQ was used to assess subclinical autistic traits and was not intended as a diagnostic instrument for autism spectrum disorder. This distinction is important because self-report autism trait measures may only partially correspond with clinician-based diagnostic assessment in adults, especially when phenotypic expression is subtle or heterogeneous (26, 27).

### 2.4 Statistical analysis

The distribution of variables was assessed using the Shapiro–Wilk test. Age showed no significant deviation from normality across the three groups, whereas AQ scores showed evidence of non-normality in at least one group. Detailed Shapiro–Wilk test results for each numerical variable by group are provided in **Supplementary Table 1**. Normally distributed continuous variables were compared among groups using one-way analysis of variance (ANOVA), whereas non-normally distributed variables were analyzed using the Kruskal-Wallis test. *Post-hoc* comparisons following a statistically significant Kruskal-Wallis test were performed using Dunn’s test with adjustment for multiple comparisons. Categorical variables were compared using the Fisher-Freeman-Halton test. Descriptive data are presented as mean ± standard deviation, median (minimum–maximum), or frequency (percentage), as appropriate. Correlations between AQ total and subscale scores were evaluated using Spearman correlation analysis. All statistical analyses were performed using IBM SPSS Statistics version 29.0.2 (IBM Corp., Armonk, NY, USA), and a two-tailed p value of <0.05 was considered statistically significant.

## 3 Results

The sociodemographic characteristics of the BD-FDR, SCH-FDR, and healthy control groups are presented in **Table 1**. No significant differences were found among the groups in terms of age, sex, marital status, alcohol use, or smoking status (all p>0.05). The groups were therefore considered comparable with respect to basic sociodemographic characteristics.

**Table 1:** Sociodemographic characteristics of the participants.

	Total (n=135)	BD-FDR (n=45)	SCH-FDR (n=45)	HCs (n=45)	p-value
Age	45.09 ± 13.46	45.09 ± 12.95	45.09 ± 14.37	45.18 ± 13.33	1^*^
Sex					
*Female*	98 (%72.6)	33 (%73.3)	33 (%73.3)	32 (%71.1)	0.963^**^
*Male*	37 (%27.4)	12 (%26.7)	12 (%26.7)	13 (%28.9)	
Marital status					
*Married*	41 (%30.4)	14 (%31.1)	14 (%31.1)	13 (%28.9)	1^**^
*Single*	94 (%69.6)	31 (%68.9)	31 (%68.9)	32 (%71.1)	
Alcohol use					
*Yes*	17 (%12.6)	6 (%13.3)	5 (%11.1)	6 (%13.3)	1^**^
*No*	118 (%87.4)	39 (%86.7)	40 (%88.9)	39 (%86.7)	
Smoking status					
*Yes*	33 (%24.4)	12 (%26.7)	14 (%31.1)	7 (%15.6)	0.203^**^
*No*	102 (%75.6)	33 (%73.3)	31 (%68.9)	38 (%84.4)	

BD-FDR, first-degree relatives of individuals with bipolar disorder; SCH-FDR, first-degree relatives of individuals with schizophrenia; HCs, healthy controls.

^*^ One Way Anova test, ^**^Fisher-Freeman Halton test.

Continuous variables are presented as mean ± standard deviation, and categorical variables as frequency (percentage).

Correlation analyses between AQ total and subscale scores are presented in **Table 2**.

**Table 2:** Correlations between total and subscale scores of the autism spectrum quotient.

AQ	Social skills	Attention switching	Attention to detail	Communication	Imagination	Total score
Social Skills	1	0.243*	0.049	0.469*	0.383*	0.715**
		0.005	0.571	<0.001	<0.001	<0.001
Attention Switching		1	-0.118	0.234*	0.215*	0.480*
			0.173	0.006	0.012	<0.001
Attention to Detail			1	0.026	0.060	0.380*
				0.768	0.491	<0.001
Communication				1	0.341*	0.660**
					<0.001	<0.001
Imagination					1	0.656**
						<0.001
Total Score						1

*p < 0.05; **p < 0.001. Spearman correlation analysis.

The internal consistency of the scale in the present sample was acceptable (Cronbach’s α=0.73). AQ total score showed significant positive correlations with all subscales. The strongest correlations were observed for social skills (r=0.715, p<0.001), communication (r=0.660, p<0.001), and imagination (r=0.656, p<0.001). Moderate correlations were also found between attention switching and total AQ score (r=0.480, p<0.001), while attention to detail showed a weaker but still significant correlation with total AQ score (r=0.380, p<0.001).

Comparisons of AQ subscale and total scores across groups are shown in **Table 3**.

**Table 3:** Comparison of autism spectrum quotient subscale and total scores across groups.

AQ	BD-FDR (n=45)	SCH- FDR (n=45)	HCs (n=45)	p-value	*Post-hoc* p-value
Social Skills	3 (0-9)	3 (0-6)	2 (0-7)	0.078^***^	–
Attention Switching	5 (1-8)	5 (2-7)	4 (1-6)	0.715^***^	–
Attention to Detail	5 (1-9)	4 (0-8)	4 (0-9)	0.431^***^	–
Communication	2 (0-8)	3 (0-6)	1 (0-5)	0.004^***^	1-2:0.659 1-3:0.123 **2-3:0.003**
Imagination	4 (1-7)	4 (0-7)	3 (0-6)	<0.001^***^	1-2:0.456 **1-3:0.016** **2-3:<0.001**
Total Score	18 (11-35)	19 (10-28)	16 (4-26)	0.002^***^	1-2:1 **1-3:0.015** **2-3:0.003**
AQ threshold					
*<26*	39 (%86.7)	37 (%82.2)	44 (%97.8)	0.045^**^	
*≥26*	6 (%13.3)	8 (%17.8)	1 (%2.2)		

1. BD-FDR, first-degree relatives of individuals with bipolar disorder; 2. SCH-FDR, first-degree relatives of individuals with schizophrenia; 3. HCs, healthy controls. ^***^Kruskal Wallis test, ^**^Fisher-Freeman Halton test.

Post-hoc comparisons were performed using Dunn’s test with adjustment for multiple comparisons; adjusted p-values are reported. Data are presented as median (minimum–maximum) or frequency (percentage).

Bold values indicate statistically significant p-values (p<0.05).

No significant group differences were observed for social skills, attention switching, or attention to detail scores (all p>0.05). Significant differences were found for communication (p=0.004), imagination (p<0.001), and total AQ score (p=0.002).

*Post-hoc* analyses showed that communication scores were significantly higher in the SCH-FDR group compared with controls (p=0.003), whereas no significant differences were found between the BD-FDR group and the other groups. For the imagination subscale, both the BD FDR group (p=0.016) and the SCH FDR group (p<0.001) had significantly higher scores than controls, with no difference between the two relative groups. Similarly, total AQ scores were significantly higher in both the BD-FDR (p=0.015) and SCH-FDR (p=0.003) groups compared with controls, while no significant difference was observed between the two relative groups.

When participants were categorized according to the AQ ≥26 threshold, significant group differences were observed (p=0.045). The proportion of individuals with AQ scores ≥26 was higher in the SCH-FDR (17.8%) and BD-FDR (13.3%) groups compared with the control group (2.2%).

## 4 Discussion

The present study found that unaffected first-degree relatives of individuals with both schizophrenia and bipolar disorder showed significantly elevated autistic traits compared with healthy controls, with group differences most prominent in total AQ scores, the Imagination subscale, and specifically in schizophrenia relatives the Communication subscale. These findings are consistent with previous reports and contribute to the literature by providing a direct comparison of autistic trait profiles in first-degree relatives of individuals with schizophrenia and bipolar disorder.

The overall pattern of elevated total AQ scores across both familial risk groups is consistent with the neurodevelopmental continuum model, which proposes that schizophrenia and bipolar disorder lie on a gradient of shared neurodevelopmental liability within which ASD also participates (2, 28). The present findings suggest that this shared vulnerability may be detectable at the familial level, even among unaffected individuals carrying familial risk for these disorders. The finding that approximately 14-18% of relatives exceeded the AQ ≥26 threshold, compared with only 2.2% of controls, is also noteworthy and consistent with previous reports documenting higher rates of subclinical autistic features in familial high-risk populations (29, 30). Although these findings do not indicate the presence of clinically diagnosable ASD, they support the view that autistic traits may represent a dimensional manifestation of familial neurodevelopmental vulnerability extending beyond clinically affected individuals.

One of the notable findings of the present study was the elevation of Communication scores, indicating greater communication-related difficulties, specifically in the SCH-FDR group. Communication difficulties in the AQ capture pragmatic language impairments, difficulties with non-literal and implied meaning, and reduced spontaneous social-communicative behavior. These features are recognized as part of the broader schizophrenia spectrum phenotype and have also been documented in unaffected relatives of individuals with schizophrenia in previous studies (31–33). This interpretation is also compatible with reports that schizophrenia relatives may show subtle impairments in higher-order language and Theory of Mind application, even in the absence of overt disorder (34). The finding that Communication scores were elevated only in schizophrenia relatives may suggest that communication-related autistic traits are more closely linked to familial vulnerability for schizophrenia than for bipolar disorder. This pattern may be consistent with the stronger overlap reported between ASD and schizophrenia within the neurodevelopmental continuum (2). From a clinical perspective, elevated Communication scores in schizophrenia relatives may represent a subtle marker of social-communicative vulnerability and may be relevant to understanding the broader social and interpersonal difficulties described across the schizophrenia spectrum. However, these communication-related differences should not necessarily be interpreted as specific to autistic traits. Social-communicative and social-cognitive difficulties are also observed in schizophrenia and bipolar disorder, and some characteristics captured by the AQ may overlap with broader psychopathological or cognitive dimensions (17). Although the participants in the present study were unaffected relatives without current or lifetime psychiatric disorders, making it less likely that these findings reflect manifestations of an overt psychiatric disorder, such overlap cannot be excluded.

Higher Imagination scores were observed in both familial risk groups, indicating greater difficulties in this domain, with no significant difference between the two relative groups. Compared with other AQ domains, the imagination subscale has received relatively less attention and appears to reflect a broader and more heterogeneous construct (35). Within the AQ framework, this subscale captures the ability to generate flexible, creative, and social imaginative content, including spontaneous narrative, perspective-taking, and mental simulation of others’ mental states (36). Previous studies have suggested that this domain may be related not only to social imagination, but also to internal representation, mental imagery, and aspects of cognitive flexibility (37). Some studies have additionally reported associations between imagination-related AQ dimensions and social-cognitive processes such as Theory of Mind performance (38). Theory of Mind difficulties have also been documented in first-degree relatives of individuals with bipolar disorder, suggesting potential links between imagination-related traits and broader social-cognitive vulnerability (39). More broadly, meta-analytic and primary-study evidence suggests that unaffected relatives of individuals with bipolar disorder may exhibit mild deficits in social cognition and selected neurocognitive domains, although such domains were not measured directly in the present study (19, 39, 40). At the same time, the psychometric structure of the imagination subscale has not been entirely consistent across studies, and its relationship with other AQ domains appears to be more variable (41). Therefore, these findings require replication in larger and independent samples.

Taken together, these findings suggest that elevated autistic traits in familial risk groups are not characterized by a uniform increase across all AQ domains. Rather, the pattern observed in the present study indicates both shared and distinct features, with imagination-related traits appearing across both relative groups and communication difficulties being more prominent among schizophrenia relatives. Consistent with this pattern, Social Skills, Attention Switching, and Attention to Detail scores did not differ significantly across groups. Previous studies have suggested that Attention to Detail may represent a relatively distinct dimension within the AQ compared with the more socially oriented subscales (40, 42). Overall, these findings suggest that autistic-like characteristics associated with familial risk for schizophrenia and bipolar disorder may be concentrated in specific AQ dimensions rather than uniformly distributed across the scale. This domain-specific pattern also suggests that the observed AQ profile may not reflect the full autistic phenotype, particularly given that restricted and repetitive behaviors constitute a core domain of ASD (5), whereas the observed group differences were confined to selected AQ dimensions. Accordingly, the present findings may be better understood as reflecting overlap in selected dimensions captured by the AQ, particularly communication and imagination, rather than indicating that a unitary autistic phenotype underlies familial vulnerability to schizophrenia and bipolar disorder. These dimensions may partly reflect broader transdiagnostic social-communicative and social-cognitive liabilities that cut across conventional diagnostic boundaries, consistent with dimensional and hierarchical approaches to psychopathology (43, 44).

One of the strengths of the present study is the inclusion of unaffected first-degree relatives of individuals with both schizophrenia and bipolar disorder within the same study design. Participants underwent a comprehensive psychiatric evaluation before inclusion. Lifetime psychiatric disorders were assessed using the Structured Clinical Interview for DSM-5 (SCID-5), and participants were also clinically evaluated for autism spectrum disorder according to DSM-5 criteria. This approach strengthened the clinical characterization of the study sample and reduced the likelihood that elevated AQ scores reflected undiagnosed psychiatric disorders or clinically manifest ASD. In addition, the comparable age and sex distributions observed across the three groups supported their sociodemographic comparability. Beyond total AQ scores, the evaluation of AQ subdomains also allowed a more detailed assessment of different autistic-like characteristics across familial risk groups.

Several limitations of the present study should be considered. First, the cross-sectional design limits causal interpretation of the findings. Second, although participants underwent a comprehensive psychiatric evaluation and clinical assessment for autism spectrum disorder, autistic traits were quantified using a self-report questionnaire rather than a standardized clinician-administered autism instrument. Therefore, subtle autism-related characteristics may not have been captured as comprehensively as with multimodal assessment approaches. Third, social cognition, neurocognitive functioning, intellectual functioning, and real-world functional outcomes were not directly evaluated, although these domains may be relevant to familial neurodevelopmental vulnerability. Measures of psychosis-spectrum characteristics, negative symptoms, and specific communication abilities were also not included. Future studies assessing these dimensions alongside autistic traits may help clarify whether the observed characteristics are relatively specific to autistic traits or partly reflect broader transdiagnostic difficulties in social-cognitive and interpersonal functioning.

## 5 Conclusion

In conclusion, the present findings suggest that autistic-like characteristics can also be observed in unaffected first-degree relatives of individuals with schizophrenia and bipolar disorder. Group differences were particularly evident in communication- and imagination-related domains, although not all AQ subdomains differed similarly across groups. These findings support the view that autistic-like features may extend beyond clinically manifest illness and may also be present at the familial level. Future longitudinal studies with larger samples are needed to determine the clinical and functional significance of autistic traits in individuals at familial risk for schizophrenia and bipolar disorder.

## Data Availability

The raw data supporting the conclusions of this article will be made available by the authors, without undue reservation.
