# Supplementary material for: Autistic traits in unaffected first-degree relatives of individuals with schizophrenia and bipolar disorder: implications for shared familial neurodevelopmental vulnerability
Source: Front Psychiatry. 2026 Sep 9;17:1933974. doi: 10.3389/fpsyt.2026.1933974 (PMC13597859; doi:10.3389/fpsyt.2026.1933974)
Supplement: Supplementary file 1 [file Table1.docx]

**Supplementary Table S1. Shapiro–Wilk Test Results for Numerical Variables Across Study Groups**

| **Variable** | **BD-FDR (n=45)** **p-value** | **SCH-FDR (n=45) p-value** | **HCs (n=45) p-value** |
| --- | --- | --- | --- |
| **Age** | 0.060 | 0.061 | 0.055 |
| **AQ Social Skills** | 0.006 | 0.020 | 0.004 |
| **AQ Attention Switching** | 0.045 | 0.004 | 0.002 |
| **AQ Attention to Detail** | 0.030 | 0.134 | 0.050 |
| **AQ Communication** | 0.002 | 0.010 | 0.002 |
| **AQ Imagination** | 0.028 | < 0.001 | 0.036 |
| **AQ Total Score** | 0.027 | 0.056 | 0.268 |

*BD-FDR: first-degree relatives of individuals with bipolar disorder; SCH-FDR: first-degree relatives of individuals with schizophrenia; HCs: healthy controls.* ***Shapiro–Wilk p-values >0.05 indicate no statistically significant evidence of deviation from normality.***
